# Monthly Summary

**Published:** 1895-05

**Authors:** 


### Monthly Summary.

"DENTAL MEDICINE."—A Manual of Dental Materia Medica and Therapeutics. By F. J. S. Gorgas, A. M., M. D., D. D. S. Fifth edition, revised and enlarged. Philadelphia: P. Blakiston, Son & Co., 1012 Walnut Street. 1895.

Evidently this is one of the most favorite, if not *the* most favorite, of the many professional labors of love in which the industrious author has engaged. Whatever Dr. Gorgas has undertaken, he has done thoroughly well; but we feel that in this work especially he has elaborated one of the best text-books in use. In addition to many new recipes, and new remedies, such as Pental, Tropacocaine, Phenosalyl, Dermatol, Eugenol-acetamid, etc., the author has made many material improvements in the general text, and, in brief, has brought the work up to the present status of dental medicine. To the dental student it is an absolute necessity. To the intelligent practitioner it is a new inspiration. To your "purely practical man," who prescribes remedies he does not understand, in pathological conditions of which he knows as little, it will even assist, and no doubt encourage, his empiricism. No doubt a great deal of the materia medica of the present day is empirical, and were every practitioner to be examined as to his knowledge of the composition, physical peculiarities, physiological effects, incomputabilities of the various drugs in daily use in dental practice, the average student could "refer back" the average dentist. It is not uncommon in these days of the free use of local anæsthetics, of whose composition the dentist knows nothing, to find the most profound ignorance as to the active absorbing power of the lympathics and capillaries of the subcutaneous cellular tissue, upon which hypodermic medication depends. It is not uncommon to find your eminently successful "practical man" as foggy on the effect of dental medicines on the circulation, how they enter, how they act when they are there, and how they are excreted, as how the milk gets into the cocoanut or the seed into the apple. A work like that before us should shame such ignorance, and make the most self-assertive "practical man" realize that he has not learned the first principles of his profession. Of course it is the bounden duty of an author to omit no important drug which has been brought before the profession. Yet, we venture to believe that the best results in practice are obtained, not by continual experimenting with the commercial fads of the pharmacist, but by confining the practice, as much as possible to a few, and those the agents which experience has proved to be most successful. We can only commend Dr. Gorgas' work as full of information and suggestion.—*Dominion Dental Journal.*

---

A NOTE ON CAPPING A TOOTH PULP.—By C. R. Taylor, D. D. S., Streator, Ill.—It is often troublesome to cap a pulp with oxyphosphate of zinc on account of that material sticking to the instruments used to convey it to the parts desired to be covered without spreading it over the whole of the cavity. By the use of the following method the troublesome part can be avoided.

Take a piece of clean writing paper of the proper size and on the paper place a sufficient quantity of the cement, soft or hard, as the case requires; having taken hold of the corner of paper with the pliers before the cement was placed on the paper, carry the paper and cement to the parts to be capped, pass a burnishing instrument against the paper and burnish the capping to its place. If it is desired to get the benefit of the sticking qualities of the cement to assist in holding the filling in the cavity, put cement on both sides of the paper, before placing it in place.

The same method can be used with the paper and chloro-percha.

Not only does the paper act as a convenient carrier but it is a splendid non-conductor. Superior to almost everything used for that purpose.—*Dental Digest.*

---

COPPER amalgam is sometimes useful for repairing rubber plates, replacing a block or broken tooth without vulcanizing.—*Dominion Dental Journal.*

THE NECESSITY OF A HIGHER ESTIMATION OF TOOTH AND MOUTH HYGIENE.—At a meeting of the Berlin Society of International Medicine Dr. P. Ritter recently made an address on the subject. He pointed out how the mouth was the starting-point and breeding-place of germs, and he had frequently seen workmen and workwomen lose their places on account of want of the incisor teeth or foul breath. Continuous toothache frequently led steady men to the brandy bottle for the relief of pain. In pregnancy the necessity for attention to the mouth and teeth was especially great, as women at these times so often suffered from tooth and face ache. Probably the cause lay in excessive nausea, thus leading them to neglect the care of their teeth, so that the secretions became acid and favored caries. Women should be recommended to brush their teeth three times a day after the third month, using some astringent fluid and an alkaline tooth powder, and a moderately hard brush. On the basis of many years' experience, he held the following to be demanded:—The appointment of experienced dentists to examine the mouths of all school children at stated intervals, the parents to be informed of the result of the examination, and left to have the treatment required carried out privately or through institutions; the appointment of dentists for the poor; delivery of addresses in the Volksschulen on the importance of the masticatory apparatus and the toilet of the mouth; dissemination of printed instructions on the toilet of the mouth to the whole of the poor population.

---

VITALITY CONDITIONED.—The native inhabitants of Greenland and Labrador, living almost exclusively on animal food, preferably that which is fat, have fine teeth almost entirely free from decay. The same is true of the Gauchos, a tribe of cattle-breeding people inhabiting the great upland plains of South America, and subsisting entirely on a meat diet. This same people, living in another part of the country, in towns, and consuming a mixed diet, suffer considerably from decay of the teeth. The Scotch people, who eat large quantities of oatmeal, and the Chinese, who live almost exclusively on a vegetable diet, have, as already mentioned, very poor teeth. The only conclusion which can be drawn from a careful examination of the foods of all nations is that vigorous people may be perfectly nourished on a great variety of food materials, while the most carefully selected diet will not maintain a high standard of vitality among a people where there is a prodigal waste of nervous energy.—Dr. J. S. Wiiliams, in *Items of Interest.*

---

THERE are some queer questions regarding the teeth not generally broached, or if mooted, not altogether settled, or if they seem to be settled, do not stay settled; such, for instance, as the following :

How much circulation is there in a healthy tooth; and what is the nature of that fluid ?

Is there any circulation in a dead tooth while still in the mouth ?

Is there any change in the constituents of a live tooth ?

Of what does the life of a tooth consist ?

What is the cause of sensitive dentine ?

Why is a tooth more subject to caries while alive than when dead ?

Why is a tooth more subject to dissolution in than out of the mouth ?

---

DR. CUNNINGHAM, of Cambridge University, says of tooth powders :—The principal action should be mechanical rather than medicinal. The powder should be very finely grained and should contain no cuttle fish powder, no powdered oyster shells, no pumice powder. It should consist of alkaline substances and contain no acid ingredients, nor such as are capable of changing to acid in the mouth. All fermentable substances such as carbo-hydrates are contra-indicated."

